# Serum Melatonin at Admission and Discharge in Schizophrenia: A Prospective Longitudinal Controlled Study

**DOI:** 10.3390/biom16101438

**Published:** 2026-10-02

**Authors:** Armando Luis Morera-Fumero, Estefanía Diaz-Mesa, Silvia Yelmo-Cruz, Pedro Abreu-González, María Lourdes Fernández-López

**Affiliations:** 1 Department of Internal Medicine, Dermatology, and Psychiatry, University of La Laguna (ULL), 38320 Santa Cruz de Tenerife, Canary Islands, Spain; ediazmes@ull.edu.es (E.D.-M.); louferna@ull.edu.es (M.L.F.-L.); 2 Department of Psychiatry, University Hospital of the Canary Islands, 38320 Tenerife, Canary Islands, Spain; silviayelmo@gmail.com; 3 Department of Basic Medical Sciences, University of La Laguna (ULL), 38320 Santa Cruz de Tenerife, Canary Islands, Spain; pabreu@ull.edu.es

**Keywords:** schizophrenia, melatonin, circadian rhythm, biomarker, psychotic relapse, longitudinal study

## Abstract

Schizophrenia is associated with circadian and neuroendocrine disturbances, but whether circulating melatonin changes with acute clinical state remains uncertain. This prospective longitudinal controlled study measured serum melatonin at 12:00 and 00:00 in 43 hospitalized patients after admission and before discharge, and during one session in 42 healthy controls. Psychopathology was assessed using the Positive and Negative Syndrome Scale (PANSS). Nonparametric comparisons used family-specific Holm adjustment; exploratory correlations used false-discovery-rate control. In comparisons without covariate adjustment, midday concentrations were higher in patients at admission and discharge (Holm *p* = 0.013 and 0.007), but the differences did not remain significant after Holm correction in exploratory models adjusted for BMI, age, and sex (29 patients and 40 controls). Median within-patient changes were −0.14 pg/mL at 12:00 (95% CI −0.27 to 0.74) and 0.00 pg./mL at 00:00 (95% CI −2.00 to 6.35); both Holm *p* = 1.000. Total PANSS decreased by a median of 14 points among 24 complete pairs. No change-PANSS correlation survived multiplicity correction. Two daily samples demonstrate a midday–midnight concentration difference but cannot characterize circadian phase, amplitude, or total secretion. No short-term melatonin change was detected during hospitalization; this does not establish equivalence or confirm or exclude trait-biomarker status. State-marker utility remains uncertain, and patient–control differences may reflect medication, smoking, or confounding by assay plate.

## 1. Introduction

Schizophrenia (SCZ) is a severe mental disorder characterized by disturbances in thought, perception, self-experience, cognition, affect, volition, and behavior [1,2]. The course commonly includes acute psychotic relapses followed by periods of partial or substantial improvement [2,3,4]. Relapses contribute to functional deterioration, hospitalization, and poorer long-term outcomes, which has prompted interest in biological processes that vary with clinical state.

The pathophysiology of SCZ involves interacting genetic, environmental, neurotransmitter, immune, oxidative, mitochondrial, neuroendocrine, and circadian mechanisms [3,4,5,6,7,8]. Melatonin (MLT) is of particular interest because it links circadian timing with neuroendocrine, metabolic, immune, and redox regulation [9,10]. Pineal MLT secretion is normally low during the day and rises at night under the control of the suprachiasmatic nucleus and the sympathetic pathway [9].

Research on MLT in SCZ has produced heterogeneous findings. Many studies reported reduced nocturnal secretion, whereas others found no group difference or higher concentrations [11,12,13,14,15,16,17,18,19,20,21,22,23]. Variation in medication exposure, biological matrix, sampling time, posture, light conditions, illness stage, and control-group selection may contribute to these discrepancies. Clinical trials of MLT in SCZ have addressed insomnia, metabolic adverse effects, tardive dyskinesia, and psychopathology [24,25,26,27].

Most previous studies were cross-sectional and therefore could not determine whether MLT changes with acute psychopathology. A state marker should vary with clinically meaningful changes in illness activity. Evaluation of a trait biomarker requires repeated observations across sustained clinical stability and different illness states; failure to detect short-term change alone cannot establish equivalence or trait status.

The present study longitudinally assessed serum MLT in patients hospitalized for an acute psychotic relapse, at admission and shortly before discharge, and compared these measurements with a healthy control group. We examined (1) patient–control differences at midday and midnight, (2) within-patient admission-to-discharge changes, (3) the day–night difference, and (4) exploratory associations with clinical variables. We hypothesized that a state-dependent marker would change alongside symptomatic improvement.

## 2. Materials and Methods

### 2.1. Participants

This prospective longitudinal controlled study included patients with SCZ consecutively admitted to the Psychiatric Inpatient Unit of the Canary Islands University Hospital because of an acute psychotic relapse. SCZ was diagnosed according to the Diagnostic and Statistical Manual of Mental Disorders, Fourth Edition, Text Revision (DSM-IV-TR) [28], using the Structured Clinical Interview for DSM-IV-TR. Two experienced psychiatrists independently evaluated the diagnosis, and only cases with complete diagnostic agreement were retained.

The initial sample comprised 48 patients. Five were excluded: one declined the second midnight sampling; one had polysubstance abuse and chronic hepatitis B; one had severe intellectual disability after bilateral frontal lobectomy; and two had diagnoses revised to delusional disorder and bipolar disorder with psychotic features. The final patient sample comprised 43 individuals.

Forty-two healthy controls were recruited from hospital and medical-school personnel. Controls had no lifetime psychiatric, neurological, or major medical disorder, normal routine laboratory results, and no first-degree relative with a psychotic disorder. The groups were comparable in sex and age at the group level. Temporal matching concerned blood-sampling dates (Section 2.3); patient–control comparisons were analyzed at the group level without matched-pair tests.

Participants were eligible if they were 18–65 years old and fluent in Spanish. Exclusion criteria included transmeridian travel during the preceding month, current benzodiazepine treatment, alcohol or substance abuse, clinically significant sleep disorders, night-shift work, pregnancy, abnormal routine biochemical or urine results, or a medical condition potentially affecting MLT secretion. Participants were instructed to avoid caffeine, alcohol, and sunglasses for 12 h before sampling, and daytime naps were not permitted on sampling days.

The protocol complied with the Declaration of Helsinki. All participants provided written informed consent. The Ethics Committee of the Canary Islands University Hospital approved the study (protocol 2008-41; 14 April 2008).

### 2.2. Clinical Assessment

Psychopathology was assessed with the validated Spanish version of the Positive and Negative Syndrome Scale (PANSS) [29,30]. The PANSS includes seven positive-symptom items, seven negative-symptom items, and 16 general-psychopathology items, each rated from 1 to 7. Subscale scores were calculated as the sums of their component items and the total score as the sum of all 30 items. A score was treated as missing when any required item was unavailable; missing items were not prorated or imputed.

The same trained psychiatrist evaluated patients within 24 h of admission and during the final 24 h before discharge. Age at illness onset, illness duration, previous psychiatric hospitalizations, body mass index (BMI), and antipsychotic treatment were extracted from clinical records. Antipsychotic doses were converted to chlorpromazine-equivalent daily doses using established conversion tables [31,32].

The analysis file contained one treatment-category variable and one chlorpromazine-equivalent dose variable reflecting prescribed treatment approximately halfway through hospitalization. These data do not represent treatment at admission or discharge; separate medication records for those time points were unavailable. Categories were risperidone alone, risperidone with other antipsychotics, and combinations of other antipsychotics. Additional agents, individual dose combinations, treatment duration, last-dose timing, adherence, and changes during hospitalization could not be reconstructed. Patients had been managed as outpatients before admission for acute psychopathological decompensation, and their preadmission treatment regimens and actual medication intake were unknown. Admission measurements were obtained in a naturalistic treatment setting and should not be interpreted as drug-naïve or verified drug-free baseline measurements; a protocolized washout was not carried out.

### 2.3. Study Design and Sampling

Serum MLT was determined at 12:00 h and 00:00 h. Patients completed sampling sessions on the day after admission and the day before discharge. Controls completed one session under the same clock-time schedule. Each healthy control was temporally matched to a patient, with blood collection performed within a maximum of seven days of the corresponding patient sampling session. Hospital-stay duration was available for 30 of 43 patients and was not imputed for the remaining 13. This clinical interval was not treated as the exact elapsed time between blood draws.

Participants remained supine for at least one hour before venipuncture to reduce postural effects on MLT levels [33]. Physical activity and emotionally stressful situations were avoided. To reduce light-induced suppression before midnight sampling, room lights were kept off throughout the two hours preceding the blood draw in both patients and healthy controls. During this period, participants also wore opaque eye masks and were advised to keep their eyes closed. Midnight collection was conducted under dim red illumination (<30 lux); daytime samples were obtained under ambient daylight. Ambient illumination was measured with a photometer. The same light-control protocol was applied to both groups.

### 2.4. Melatonin Assay

Venous blood was collected into serum tubes, allowed to clot, centrifuged at 3000 rpm for 10 min, and stored at −70 °C. Serum MLT was measured with a competitive ELISA (IBL International GmbH, Hamburg, Germany). Absorbance was measured at 450 nm, and concentrations were calculated from a four-parameter logistic calibration curve. The reported analytical sensitivity was 0.17 pg./mL; intra-assay and inter-assay coefficients of variation were 13.2% and 16.5%, respectively.

Samples were analyzed in August 2010 in one laboratory by the same technician, blinded to group, visit, and sampling time. Serum was thawed once, immediately before analysis. Based on recruitment from January to June 2009 and assay in August 2010, storage of admission samples was approximately 14–19 months, and discharge specimens had correspondingly shorter intervals. Samples were not assayed in duplicate because the available serum volume was insufficient. Each patient’s paired admission and discharge specimens were analyzed on the same ELISA plate. Healthy-control specimens were analyzed on a different plate. This allocation reduces between-plate variation for paired patient comparisons but confounds patient–control status with plate allocation.

### 2.5. Statistical Analysis

Data were entered into an individual-level dataset containing 43 patients and 42 controls. Analyses used the SPSS analysis file. Missing data were handled by available-case analyses for descriptive and between-group comparisons and complete pairs for longitudinal comparisons. Standard PANSS sums were reconstructed from the item-level variables as described above. No missing values were imputed.

Continuous variables were inspected graphically and with the Shapiro–Wilk test. Because all six MLT variables deviated from normality, MLT is summarized by median and interquartile range (IQR). Patient–control comparisons used two-sided Mann–Whitney U tests; admission-to-discharge and midday-to-midnight comparisons used two-sided Wilcoxon signed-rank tests. Rank-biserial correlations quantify effect size. Sex and available-case smoking distributions were compared with two-sided Fisher exact tests. Within-patient change was defined as discharge minus admission. Pointwise 95% percentile bootstrap confidence intervals (CIs) for the median of the individual MLT changes used 50,000 resamples of patients, preserving paired observations. Sample-characteristic comparisons and sex comparisons of MLT were reported with unadjusted *p* values and interpreted descriptively.

The main state-marker comparisons were the two admission-to-discharge MLT changes. No dated analysis plan or preregistration was available to verify prospective specification; analyses already presented in the manuscript are therefore termed main study comparisons, and additional adjustment models and change correlations are termed reviewer-requested exploratory analyses. Holm adjustment was applied separately to the two longitudinal MLT comparisons, four patient–control MLT comparisons, three midday–midnight comparisons, and four secondary PANSS changes. These separate families do not provide study-wide family-wise error control (Table S4).

The exploratory correlation family comprised each of the four patient MLT measures crossed with 12 variables: age, BMI, chlorpromazine-equivalent dose, age at illness onset, illness duration, previous hospitalizations, and PANSS positive, negative, and general scores at both admission and discharge. Benjamini–Hochberg (BH) correction was applied jointly to these 48 Spearman tests. Eight additional Spearman correlations related changes in MLT at the two sampling times to changes in positive, negative, general, and total PANSS. Two-sided permutation *p* values used 49,999 permutations, and pointwise 95% percentile CIs used 20,000 paired bootstrap resamples. These eight tests formed a separate BH family; pooling all 56 correlations was a further sensitivity check. Bootstrap and permutation procedures used reproducible pseudorandom sampling with an initial seed of 4564756.

Four exploratory linear-regression models, one for each patient visit and sampling time, regressed natural-log MLT on patient/control group, BMI, age, and sex. Exponentiated group coefficients estimate adjusted patient/control geometric-mean ratios. HC3 heteroskedasticity-robust standard errors and t-based inference were used. Each model included each control once; control measurements were not treated as longitudinal repeats. An additional set of four models also adjusted for smoking status. Each four-model set was separately Holm-adjusted. Complete-case sample sizes are reported with each analysis. Confidence intervals are pointwise and are not multiplicity-adjusted. Statistical significance required a two-sided adjusted *p* or q < 0.05 within the relevant family. Analyses used Python.

## 3. Results

### 3.1. Sample Characteristics and Data Completeness

The analyzed sample comprised 43 patients and 42 controls. Sex distributions were comparable (33 men and 10 women among patients; 33 men and nine women among controls; Fisher’s exact *p* = 1.000). Age did not differ between groups. BMI was higher in patients (U = 784.0, *p* = 0.024; rank-biserial correlation = 0.319). The mean chlorpromazine-equivalent dose prescribed approximately halfway through hospitalization was 757.9 (SD 443.5) mg/day. Mean age at illness onset was 22.5 (SD 7.1) years, mean illness duration was 14.7 (SD 11.4) years, and the mean number of previous hospitalizations was 5.3 (SD 4.6) (Table 1).

At approximately the midpoint of hospitalization, prescribed treatment for all 43 patients was risperidone alone in 10 (23.3%), risperidone with other antipsychotics in 16 (37.2%), and combinations of other antipsychotics in 17 (39.5%). Thus, 33 patients (76.7%) had a combination-treatment classification. Hospitalization duration was available for 30 patients (69.8%): median 20.5 days, IQR 14.25–26.75, range 2–51. Thirteen values were missing and were not imputed.

Among patients, 23/43 (53.5%) were smokers and 20/43 (46.5%) were nonsmokers. Among the 33 controls seven (21.2%) were smokers and 26 (78.8%) were nonsmokers.

### 3.2. Melatonin Concentrations

Patients had higher midday MLT than controls at admission (U = 1228.5, unadjusted *p* = 0.004, Holm-adjusted *p* = 0.013, rank-biserial correlation = 0.360) and discharge (U = 1261.5, unadjusted *p* = 0.002, adjusted *p* = 0.007, rank-biserial correlation = 0.397). Nighttime MLT did not differ significantly between patients and controls at admission or discharge (Table 2).

No admission-to-discharge MLT change was detected at 12:00 (W = 422.0, raw *p* = 0.538) or 00:00 (W = 416.0, raw *p* = 0.657); both Holm *p* values were 1.000. The median of the individual changes was −0.14 pg./mL at 12:00 (95% bootstrap CI −0.27 to 0.74) and 0.00 pg./mL at 00:00 (95% CI −2.00 to 6.35), with 43 pairs at each time. Midnight concentrations exceeded midday concentrations at both patient visits and in controls (all Holm *p* < 0.001; Table 3).

Exploratory sex comparisons, reported with unadjusted *p* values, detected no difference in MLT among patients at any visit/time combination (all *p* ≥ 0.645) or among controls at 12:00 or 00:00 (both *p* ≥ 0.540). These comparisons were interpreted descriptively.

The BMI-, age-, and sex-adjusted models included 29 patients and 40 controls (*n* = 69). Fourteen patients lacked BMI, and two distinct controls were excluded, one for missing age and one for missing BMI. At 12:00, adjusted patient/control ratios were 1.59 at admission (95% CI 1.055–2.401; raw *p* = 0.027; Holm *p* = 0.082) and 1.69 at discharge (95% CI 1.093–2.605; raw *p* = 0.019; Holm *p* = 0.076). Neither midday comparison met the 0.05 threshold after correction across the four models; the 00:00 comparisons were also nonsignificant after correction (Table S1).

The models additionally adjusted for smoking included 29 patients and 33 controls (*n* = 62). At 12:00, ratios were 1.60 at admission (95% CI 1.002–2.555; raw *p* = 0.049; Holm *p* = 0.194) and 1.68 at discharge (95% CI 1.004–2.815; raw *p* = 0.048; Holm *p* = 0.194). At 00:00, ratios were 1.27 (95% CI 0.75–2.13; Holm *p* = 0.366) and 1.45 (95% CI 0.92–2.27; Holm *p* = 0.211), respectively (Table S2). No comparison survived Holm correction. Because the complete-case sample differed from that of the models without smoking, these estimates do not isolate the effect of smoking adjustment alone.

### 3.3. Clinical Change and Exploratory Correlations

PANSS scores decreased between admission and discharge in complete paired observations: 41 pairs for positive symptoms, 24 for negative symptoms, 41 for general psychopathology, and 24 for total PANSS (Table 4). All four changes remained significant after Holm correction. Total PANSS decreased by a median of 14 points among the 24 complete pairs (W = 4.5; Holm *p* < 0.001; rank-biserial correlation = −0.964).

In the explicitly reconstructed 48-test correlation family, illness duration showed an inverse association with admission 12:00 MLT (rho = −0.313, *p* = 0.041). No association remained significant after BH correction (minimum q = 0.804). None of the eight MLT-change-PANSS-change correlations survived their separate BH correction (Table S3). Total PANSS change correlated with MLT change at rho = −0.270 at 12:00 (95% CI −0.659 to 0.194; q = 0.496) and rho = −0.169 at 00:00 (95% CI −0.575 to 0.272; q = 0.683), with *n* = 24 for each. Their wide intervals do not exclude associations. Pooling all 56 correlations left every change correlation nonsignificant (minimum q among the eight change correlations = 0.760).

## 4. Discussion

The principal result of our research is that no short-term change in serum MLT was detected during inpatient clinical improvement. The daytime patient–control difference requires caution because the exploratory covariate-adjusted comparisons did not survive their multiplicity correction, and the group was confounded by the ELISA plate. These observations leave both the physiological explanation and the clinical usefulness of MLT uncertain.

Higher daytime MLT has been reported in psychiatric patients receiving chlorpromazine [17,34] and in morning samples from women with SCZ [23]. These studies differ from ours in diagnostic composition, sex distribution and sampling time. The present daytime finding may reflect the clinical and treatment context and cannot be generalized to increased 24 h MLT output.

The absence of a detected midnight group difference contrasts with reports of reduced nocturnal MLT in SCZ [11,12,14,16,22,35], but other studies found no difference or more complex temporal abnormalities [19,20,21,22,35,36]. Variation in illness stage, medication, biological matrix, season, posture, light exposure, assay, and control selection may contribute to this heterogeneity. Measurements at 08:00, 12:00, 00:00, and 02:00 sample different portions of the MLT profile and are not interchangeable. Although MLT can show seasonal variation, each HC was sampled within a maximum of seven days of the corresponding patient sampling session. This temporal proximity reduced the likelihood of a substantial seasonal imbalance between patients and controls.

The difference between 12:00 and 00:00 does not characterize circadian phase, amplitude, acrophase, dim-light MLT onset, or total secretion [35,37]. Rao et al. [35] identified a possible phase advance using repeated 24 h sampling and cosinor analysis, illustrating how a difference between two clock times can coexist with altered phase. Our data establish only a midday–midnight concentration difference under the reported collection protocol, not normal circadian organization.

Previous studies have also described dissociations between symptom improvement and MLT, including persistence of reduced profiles after antipsychotic treatment [21,38]. Maiti et al. [39] reported reductions in 02:00 serum MLT after haloperidol or risperidone, and Yao et al. [40] reported lower MLT in first-episode patients after four weeks of antipsychotic treatment than in healthy controls, but no significant within-patient change from pretreatment levels. Mann et al. [41] found no change in nocturnal MLT output after olanzapine despite improvement in PANSS. Drug, sampling time, treatment duration, and prior exposure may therefore influence longitudinal findings.

The nonsignificant longitudinal tests do not establish equivalence or exclude clinically relevant effects. No clinically justified equivalence margin or biomarker-performance threshold was defined. The change intervals and imprecise change-PANSS associations limit inference about state-marker utility. Discharge represents short-term improvement, and this analysis does not evaluate sustained remission, repeated stable-state measurements, or recurrence across relapses. It therefore neither confirms nor rules out trait-biomarker status.

The corrected correlation results provide no statistically robust association with clinical variables, consistent with the variable relationships reported previously [19,21,35,36,38]. However, the limited numbers of complete PANSS pairs, especially for negative and total scores, reduce precision. Nonsignificant correlations should not be interpreted as proof that MLT is independent of symptom severity or clinical improvement.

Medication remains a potential confounder. Earlier work described increased MLT with chlorpromazine and different responses to other agents [17,34], while subsequent studies reported absent effects or reductions after treatment [21,38,39,41]. Most patients had a combination-treatment classification based on prescriptions approximately halfway through hospitalization. These records do not represent treatment at admission or discharge, and individual additional agents and admission-to-discharge changes were unavailable. Chlorpromazine-equivalent dose cannot capture receptor pharmacology, combinations, treatment duration, adherence, or last-dose timing; its nonsignificant correlation with MLT cannot exclude medication effects. Preadmission treatment and actual medication intake were unknown in these previously ambulatory patients admitted with acute psychopathological decompensation. The admission assessment was potentially influenced by residual or ongoing medication and cannot isolate untreated schizophrenia.

The covariate analyses qualify the apparent daytime elevation. BMI differed between groups, and the estimates adjusted for BMI, age, and sex were imprecise and did not remain significant after Holm correction. Earlier findings also indicate that body-size adjustment can attenuate MLT group differences [11]. Complete-case adjustment excluded approximately one-third of patients because of missing BMI, introducing possible selection bias as well as loss of precision. The small number of women also limits inference about sex-specific effects [23,35].

MLT has antioxidant, mitochondrial, anti-inflammatory, and immunomodulatory functions [9,10,42,43], but this study cannot determine whether these pathways explain the observed concentrations. Altered circadian phase, medication effects, and responses to oxidative or inflammatory processes remain hypotheses. Information on polypharmacy and treatment-related inflammatory changes in other studies [44,45,46] further illustrates why a single dose-equivalent variable cannot resolve pharmacological heterogeneity.

Smoking and habitual caffeine exposure remain potential sources of confounding. Smoking was more frequent among records with verified status, but nine unresolved control linkages prevent a complete prevalence comparison. The smoking-adjusted models did not produce significant group comparisons after Holm correction and used a different complete-case sample. Tobacco smoke induces hepatic CYP1A2 and increases the metabolism of exogenous MLT. Ursing et al. found reduced exposure to orally administered MLT during smoking, but no significant difference in endogenous nocturnal MLT concentrations compared with seven days of abstinence [47]. Thus, increased metabolism of exogenous MLT does not establish a reduction in endogenous concentrations in the present study. Missing patient cigarette consumption, unrecorded smoking abstinence, and unquantified habitual caffeine intake and adherence to the 12 h restriction limit further adjustment.

Strengths include complete MLT measurements at both patient visits, assessment of clinically documented improvement, a healthy comparison group, and paired admission/discharge assays on the same plate. However, separate plates for patients and controls make group and plate effects inseparable. Neither covariate adjustment nor use of one reagent batch can remove this confounding. Consequently, between-group findings warrant greater caution than the paired within-patient comparisons.

Other limitations include the modest single-center sample and incomplete clinical and covariate data. Partial or complete nonadherence to outpatient antipsychotic treatment is an important risk factor for psychotic relapse [48]. In this cohort, however, the extent of any reduction or discontinuation before admission was unknown, so neither the individual cause of relapse nor prior drug exposure could be determined. The same light-control protocol was applied to both groups, including room lights off, opaque eye masks and advice to keep the eyes closed during the two hours before midnight sampling. Equivalent retinal exposure was not established because continuous light measurements, adherence logs and records of interruptions were unavailable. The study chronology and storage conditions are described in Section 2.4. This verified chronology does not itself establish MLT stability during storage, so storage-related bias cannot be excluded. Samples were not assayed in duplicate because the available serum volume was insufficient. The short observation window and two daily samples further constrain biomarker and circadian interpretations. Future studies require denser sampling, documented medication and light exposure, balanced assay allocation, and repeated assessments across remission and relapse.

## 5. Conclusions

No short-term change in serum MLT was detected during hospitalization despite improvement in PANSS scores. This does not establish equivalence, and state-marker utility remains uncertain. The findings neither confirm nor exclude trait-biomarker status. The daytime patient–control difference remains exploratory because it did not survive multiplicity correction in adjusted analyses and may reflect medication, smoking, or assay-plate effects.

## Figures and Tables

**Table 1:** Demographic and clinical characteristics.

Variable	Patients	Controls	Test	*p*
Age, years	37.6 (10.8) *n* = 43	38.2 (11.1) *n* = 41	U = 857.0	0.830
Men/women, *n*	33/10	33/9	Fisher exact	1.000
BMI, kg/m^2^	29.3 (7.5) *n* = 29	25.4 (4.4) *n* = 41	U = 784.0	0.024
Age at illness onset, years	22.5 (7.1)	—	—	—
Illness duration, years	14.7 (11.4)	—	—	—
Previous hospitalizations, *n*	5.3 (4.6)	—	—	—
Chlorpromazine equivalents, mg/day	757.9 (443.5)	—	—	—
Risperidone alone, *n* (%)	10/43 (23.3)	—	—	—
Risperidone plus other antipsychotics, *n* (%)	16/43 (37.2)	—	—	—
Combinations of other antipsychotics, *n* (%)	17/43 (39.5)	—	—	—
Hospital stay, days, median [IQR]	20.5 [14.25–26.75] *n* = 30	—	—	—
Current smokers, *n*/available *n* (%)	23/43 (53.5)	7/33 (21.2)	Fisher exact	0.005

BMI, body mass index; SD, standard deviation; IQR, interquartile range; U, Mann–Whitney U statistic. Values are mean (SD) unless indicated. Group sizes are 43 patients and 42 controls; available denominators are specified for incomplete or subgroup variables. Age was missing in one control; BMI was missing in 14 patients and one control. The 30 observed hospital stays ranged from 2 to 51 days; 13 stays were missing. Smoking was unknown for nine controls, who were excluded from its Fisher exact comparison. Treatment categories and chlorpromazine-equivalent doses reflect prescriptions approximately halfway through hospitalization and do not represent treatment at admission or discharge. —, not applicable or unavailable. The *p* values in this descriptive table are unadjusted for multiplicity.

**Table 2:** Serum melatonin concentrations in patients and controls.

Visit and Time	Patients Median [IQR]	Controls Median [IQR]	U	Raw *p*	Holm *p*	r_rb
Admission 12:00	5.11 [2.98–7.64]	2.79 [1.56–5.05]	1228.5	0.004	0.013	0.360
Admission 00:00	33.22 [16.58–89.46]	26.84 [19.40–45.59]	978.0	0.513	0.513	0.083
Discharge 12:00	5.60 [2.93–7.76]	2.79 [1.56–5.05]	1261.5	0.002	0.007	0.397
Discharge 00:00	34.81 [17.02–92.53]	26.84 [19.40–45.59]	1033.0	0.255	0.510	0.144

IQR, interquartile range; U, Mann–Whitney U statistic; Holm *p*, Holm-adjusted *p* value; r_rb, rank-biserial correlation. Concentrations are in pg/mL; *n* = 43 patients and 42 controls in each comparison. These tests are unadjusted for covariates; Holm correction covers the four group comparisons. The same single control sampling session supplies the cross-sectional comparator for each visit. Ratios from covariate-adjusted exploratory models are reported in Table S1 and Table S2.

**Table 3:** Within-participant melatonin comparisons.

Comparison	*n*	Median Change [95% CI]	W	Raw *p*	Holm *p*	r_rb
Patients 12:00 Admission to discharge	43	−0.14 [−0.27 to 0.74]	422.0	0.538	1.000	0.108
Patients 00:00 Admission to discharge	43	0.00 [−2.00 to 6.35]	416.0	0.657	1.000	0.079
Patients at admission 12:00 versus 00:00	43	—	3.0	<0.001	<0.001	0.994
Patients at discharge 12:00 versus 00:00	43	—	19.0	<0.001	<0.001	0.960
Controls 12:00 versus 00:00	42	—	0.0	<0.001	<0.001	1.000

W, Wilcoxon signed-rank statistic; r_rb, rank-biserial correlation; CI, confidence interval. Changes and CIs are in pg/mL. Longitudinal change is discharge minus admission; its estimate is the median of individual paired differences, not the difference between visit medians. Pointwise percentile CIs used 50,000 patient resamples. Holm correction was applied separately to the two longitudinal and three midday–midnight tests. Positive r_rb denotes higher discharge than admission concentrations for longitudinal comparisons, or higher 00:00 than 12:00 concentrations for diurnal comparisons. —, change estimate and CI not reported for the diurnal comparisons.

**Table 4:** Change in PANSS scores from admission to discharge.

PANSS Score	*n* Paired	Admission Median [IQR]	Discharge Median [IQR]	Median Change	W	Holm *p*	r_rb
Positive	41	19.00 [15.50–23.00]	10.00 [7.00–14.75]	−8.0	23.5	<0.001	−0.943
Negative	24	12.00 [9.00–18.75]	11.00 [9.00–13.00]	−2.0	30.0	0.009	−0.684
General	41	24.00 [21.00–28.00]	19.00 [17.00–23.00]	−5.0	79.0	<0.001	−0.775
Total	24	55.00 [50.25–64.75]	42.00 [37.00–46.00]	−14.0	4.5	<0.001	−0.964

IQR, interquartile range; PANSS, Positive and Negative Syndrome Scale; W, Wilcoxon signed-rank statistic; r_rb, rank-biserial correlation. Admission and discharge medians summarize available observations at each visit. The *n* column refers specifically to complete admission/discharge pairs used for the change analyses. Median change is the median of individual discharge-minus-admission differences. Holm *p* values are adjusted across the four PANSS outcomes. Negative effects denote lower discharge scores. Scores with any missing required item were not calculated.

## Data Availability

The data are not publicly available because they contain sensitive clinical information and are subject to ethical, privacy, and institutional restrictions. Reasonable inquiries may be directed to the corresponding author and will be considered subject to the applicable approvals.

## Supplementary Materials

The following supporting information can be downloaded at: https://www.mdpi.com/article/10.3390/biom16101438/s1. Table S1: Patient/control geometric-mean ratios adjusted for BMI, age and sex; Table S2: Patient/control geometric-mean ratios additionally adjusted for smoking; Table S3: Spearman correlations between changes in melatonin and PANSS; Table S4: Analysis status and multiplicity families.

## Abbreviations

BH, Benjamini–Hochberg; BMI, body mass index; CI, confidence interval; ELISA, enzyme-linked immunosorbent assay; FDR, false discovery rate; HC3, heteroskedasticity-consistent covariance estimator type 3; IQR, interquartile range; MLT, melatonin; PANSS, Positive and Negative Syndrome Scale; SCZ, schizophrenia.
