# Supplementary material for: Serum Melatonin at Admission and Discharge in Schizophrenia: A Prospective Longitudinal Controlled Study
Source: Biomolecules. 2026 Oct 2;16(10):1438. doi: 10.3390/biom16101438 (PMC13702438; doi:10.3390/biom16101438)
Supplement: Supplementary file 1 [file biomolecules-16-01438-s001.zip › biomolecules-4564756-supplementary.pdf]

Supplementary Analyses

Table S1. Patient/control geometric-mean ratios adjusted for BMI, age and sex.

Visit and time	n P / C	Ratio	95% CI	Raw p	Holm p
Admission 12:00	29 / 40	1.59	1.055 to 2.401	0.027	0.082
Admission 00:00	29 / 40	1.15	0.667 to 1.972	0.616	0.616
Discharge 12:00	29 / 40	1.69	1.093 to 2.605	0.019	0.076
Discharge 00:00	29 / 40	1.54	0.996 to 2.389	0.052	0.104

P, patients; C, controls; CI, confidence interval. All four log-linear models included 29 patients and 40 controls and used HC3 robust standard errors with t-based inference. Ratios above 1 indicate higher estimated concentrations in patients. Holm correction covers these four group comparisons. CIs are pointwise; an interval excluding 1 can coexist with Holm $p > 0.05$. No missing covariates were imputed.

Table S2. Patient/control geometric-mean ratios additionally adjusted for smoking.

Visit and time	n P / C	Ratio	95% CI	Holm p
Admission 12:00	29 / 33	1.60	1.002 to 2.555	0.194
Admission 00:00	29 / 33	1.27	0.75 to 2.13	0.366
Discharge 12:00	29 / 33	1.68	1.004 to 2.815	0.194
Discharge 00:00	29 / 33	1.45	0.92 to 2.27	0.211

All four exploratory models included BMI, age, sex and verified smoking status; HC3 robust standard errors and t-based inference were used. Holm correction is applied within this separate four-model family. CIs are pointwise. Raw p values for the 12:00 comparisons were 0.049 at admission and 0.048 at discharge. Nine controls with unresolved smoking linkage were excluded. Because the sample differs from Table S1, the two model sets do not isolate the contribution of smoking adjustment alone.

Table S3. Spearman correlations between changes in melatonin and PANSS.

Time	PANSS	n	rho	95% CI	p	BH q
12:00	Total	24	-0.270	-0.659 to 0.194	0.203	0.496
12:00	Positive	41	0.087	-0.241 to 0.421	0.589	0.785
12:00	Negative	24	-0.332	-0.672 to 0.100	0.113	0.496
12:00	General	41	-0.214	-0.459 to 0.057	0.181	0.496
00:00	Total	24	-0.169	-0.575 to 0.272	0.427	0.683
00:00	Positive	41	-0.002	-0.340 to 0.324	0.988	0.998
00:00	Negative	24	-0.245	-0.653 to 0.246	0.248	0.496
00:00	General	41	0.001	-0.345 to 0.348	0.998	0.998

All changes are discharge minus admission. Positive rho indicates changes in the same direction and negative rho indicates opposite directions. Two-sided permutation p values used 49,999 permutations; pointwise 95% percentile CIs used 20,000 paired bootstrap resamples. BH correction covers the eight tests shown. The initial pseudorandom seed was 4564756. Pooling these tests with the 48-test clinical correlation family left every change correlation nonsignificant (minimum q among these eight tests = 0.760).

Table S4. Analysis status and multiplicity families.

Comparison family	Tests	Status	Correction
Admission-to-discharge MLT changes	2	Main state-marker comparisons	Holm
Patient/control MLT comparisons	4	Main group comparisons	Holm
Midday/midnight differences	3	Main diurnal comparisons	Holm
PANSS positive, negative, general and total changes	4	Secondary clinical outcomes	Holm
Four MLT measures × 12 clinical variables	48	Reconstructed exploratory family	BH
MLT change × PANSS change	8	Reviewer-requested exploratory	BH
Group models adjusted for BMI, age and sex	4	Reviewer-requested exploratory	Holm
Group models additionally adjusted for smoking	4	Reviewer-requested exploratory	Holm

MLT, melatonin; PANSS, Positive and Negative Syndrome Scale; BH, Benjamini–Hochberg false-discovery-rate procedure. Corrections are applied within, not across, the listed families; no study-wide family-wise error guarantee is claimed. A dated prospective analysis plan was unavailable. The 12 variables defining the 48-test family are listed in Section 2.5. Pooling the 48 and eight correlation tests is an additional sensitivity analysis. All 95% CIs are pointwise.
